# A human plasma derived supplement preserves function of human vascular cells in absence of fetal bovine serum

**DOI:** 10.1186/s13578-017-0164-4

**Published:** 2017-08-14

**Authors:** C. Castells-Sala, J. Martorell, M. Balcells

**Affiliations:** 1 IQS School of Engineering, Universitat Ramon Llull, Barcelona, Spain; 2 Institute for Medical Engineering and Science, Massachusetts Institute of Technology, Cambridge, MA USA

## Abstract

Current techniques for cell culture routinely use animal-derived components. Fetal bovine serum (FBS) is the most widely applied supplement, but it often displays significant batch-to-batch variations and is generally not suitable for clinical applications in humans. A robust and xeno-free alternative to FBS is of high interest for cellular therapies, from early in vitro testing to clinical trials in human subjects. In the current work, a highly consistent human plasma derived supplement (SCC) has been tested, as a potential substitute of FBS in primary human vascular cells culture. Our results show that SCC is able to support proliferation, preserve cellular morphology and potentiate functionality analogously to FBS. We conclude that SCC is a viable substitute of FBS for culture and expansion of cells in advanced therapies using human vascular cells and fibroblasts.

## Introduction

Dear Editor,

The development of cellular therapies for the treatment of malignant and non-malignant diseases has grown exponentially over the last decade. Nevertheless, only a few products for cell therapy such as Carticel, Azficel-T and ALLOCORD have been approved for medical use. The road from bench top to market of human grafts, devices and cell therapy products can be long; these products usually undergo extensive in vitro testing prior to preclinical studies in established animal models and final implantation in human subjects. Additionally, in vitro cellular expansion is often necessary to attain the required number of cells prior to clinical use. In-depth cellular characterization is extremely important in order to establish the most appropriate environment for expansion, analysis and implantation. It is widely known that environmental conditions affect cellular phenotype and function [1, 2] and hence choosing the right milieu is key to a successful therapy. Fetal bovine serum (FBS), in particular, which is rich in growth factors (GF) and low molecular weight molecules like hormones, and has been demonstrated to possess low levels of antibodies, is a common supplement due to its widespread availability and easy storage [7]. It has been previously reported, however, that FBS introduces a great deal of inter-batch variability, which means that depending on the FBS batch used, cells may respond differently to identical stimuli [7]. Since its introduction, cell culture has remained mostly unaltered. Current techniques for cell culture routinely use animal-derived components. Theoretical risks of using human cells cultured with animal-derived reagents for transplantation include severe anaphylaxis, presence of animal pathogens and immune reactions due to presence of xenogeneic antigens [1, 3]. Most available cell lines have been directly or indirectly exposed to products of animal origin during their derivation and/or propagation in vitro, which limits their clinical application [3, 4].

Good manufacturing practices (GMPs) are essential to provide cells with well-defined characteristics that are safe for the patient. GMP quality, defined by both the European Medicine Agency and the Food and Drug Administration, is a requirement for clinical grade cells that offer optimal defined quality and safety for cellular transplantation [5]. It covers both manufacturing and testing of the final product, traceability of raw materials and validated standard operating procedures (SOPs). These quality and safety requirements for cell-based therapeutic products prompted the scientific community to consider removing xeno-derived components from every step of the production of cells; i.e. derivation, passaging, expansion and cryopreservation procedures [3, 4].

FBS has been used in cellular transplantation only in life-and-death situations. Macchiarini et al. documented a case in which a tissue-engineered airway was implanted, using a donor with blood type O and where major histocompatibility complexes were thoroughly removed using exhaustive detergent methods [6]. The safety and quality of the transplanted cells may be significantly enhanced by replacing FBS and other animal-derived cell culture reagents with allogenic human serum (HS) or a defined serum-free (SF) or xeno-free (XF) media formulation. Human serum has been used to replace FBS in cell culture with success limited to a few encouraging cases [7]. Richards and Ellerström (in 2002 and 2006, respectively) derived human embryonic stem cells lines (hESC) using culture medium containing human serum. Ellerström was able to propagate hESC in HS-containing culture medium in an undifferentiated state for over 20 passages. Additionally, pooled allogenic HS, platelet-rich plasma or human platelet lysate have been reported to support equally higher proliferation rates and multilineage differentiation capacity of bone marrow mesenchymal stem cells and adipose stem cells [1]. Human sera-based supplements have been previously tested in the culture of human mesenchymal stem cells as potential substitutes for FBS [8]. The current consensus is that human-derived serum supplements are a safe alternative to FBS in stem-cell based therapies. However, in the few successful cases published, the serum was pooled individually for each patient.

The xeno-free human plasma derived supplement SCC, under development at Grifols, is a freeze-dried preparation obtained from human plasma through cold-ethanol industrial fractionation manufactured under good manufacturing practice (GMP) rules. Fractionation plasma pools contain donations from over 1000 different healthy donors, ensuring that SCC biological properties are consistent from lot to lot. Overall, SCC’s advantage is that, despite being from human origin, it possesses a well-defined composition, no batch to batch variability and no xenogeneic traces, which are the main concerns raised by the FDA to approve sera for stem-cell based therapies.

Here, we explore and characterize the role played by a human plasma derived xeno free supplement (SCC) as an alternative to FBS in the regulation of cell growth, morphology, phenotype and activity in two-dimensional cellular cultures. Our results suggest that SCC effectively guarantees cellular proliferation, morphology, phenotype, and activity in three highly relevant cell types that can be used to recapitulate the arterial structure in research in cardiovascular diseases: human umbilical vein endothelial cells (hUVEC), human aortic smooth muscle cells (hAoSMC) and normal human dermal fibroblasts (NHDF).

## Results

### Proliferation

Proliferation capacity of human umbilical vein endothelial cells (hUVEC), human aortic smooth muscle cells (hAoSMC), and normal human dermal fibroblasts (NHDF) was analyzed in medium supplemented with SCC or FBS at different concentrations (5 and 10%). Cells were trypsinized and cultured in a 48 well-plate at the cell density recommended by the supplier (hUVEC—6500 cells/cm^2^; hAoSMC—7500 cells/cm^2^ and NHDF—4000 cells/cm^2^) and exposed to the corresponding serum (FBS or SCC) for at least two doubling times prior to any experimentation in order to assure proper acclimation to the serum.

**Fig. 1:**
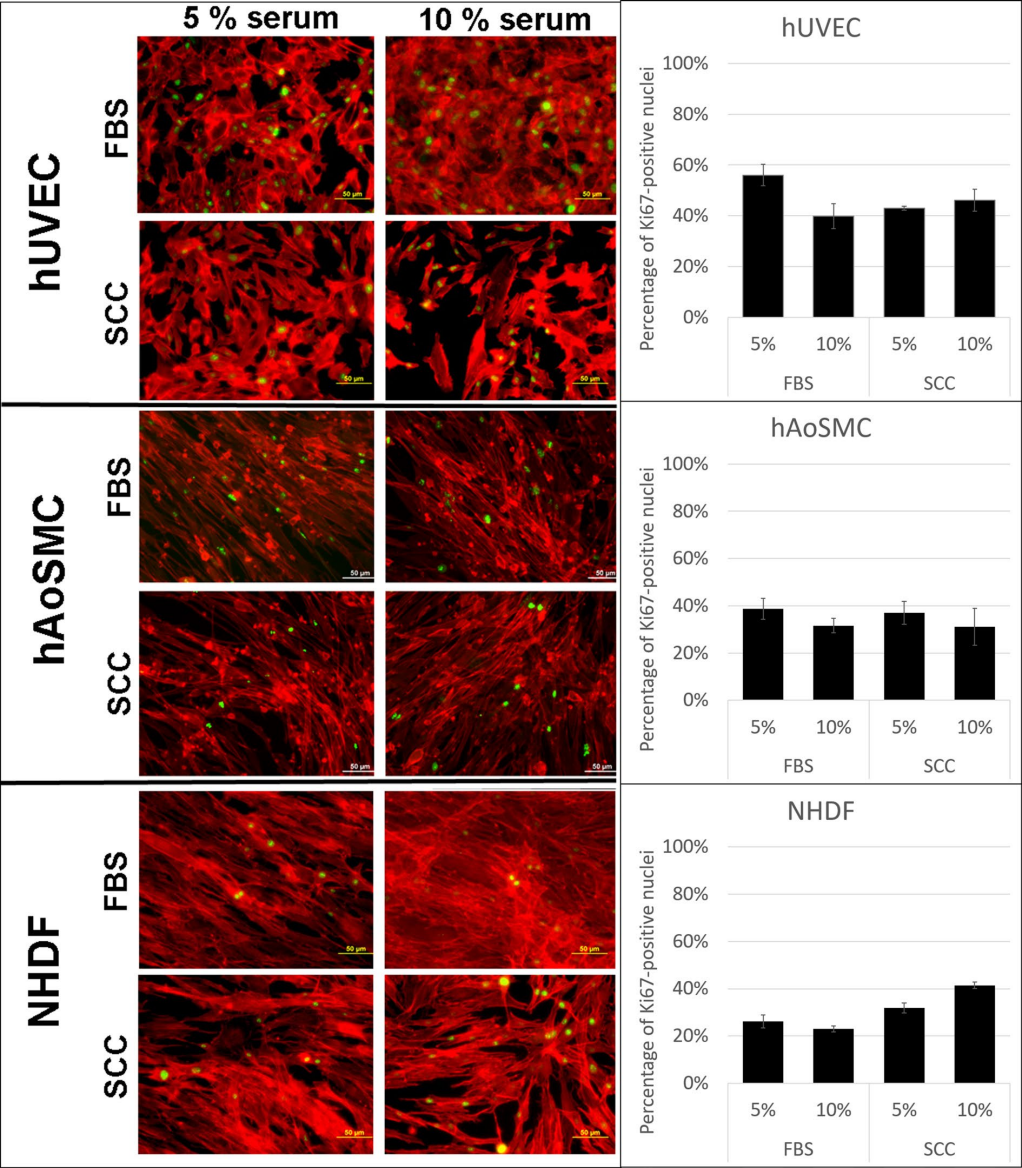
Proliferation analysis by immunofluorescence staining of Ki-67 after 36 h of culture. Ki67 expression (*green*) can be observed in the nuclei of every cell type in FBS- and SCC-supplemented media. Cells were counterstained for actin filaments using phalloidin-rhodamine. The number of Ki67-positive nuclei was divided by the number of total nuclei to give an estimate of cells proliferating. Results are presented as mean ± standard deviation obtained from three independent values per group. No significant differences were observed between groups after nonparametric Kruskal–Wallis test, followed by a Scheffé posthoc analysis

Ki-67, a nuclear transcription factor crucial for cellular proliferation, was stained in order to estimate cellular proliferation (Fig. 1). We measured the percentage of nuclei that stained positively for Ki-67, showing that hUVEC, hNDF and hAoSMC are capable to proliferate in both supplemented media either at 5 and 10% FBS and SCC.

**Fig. 2:**
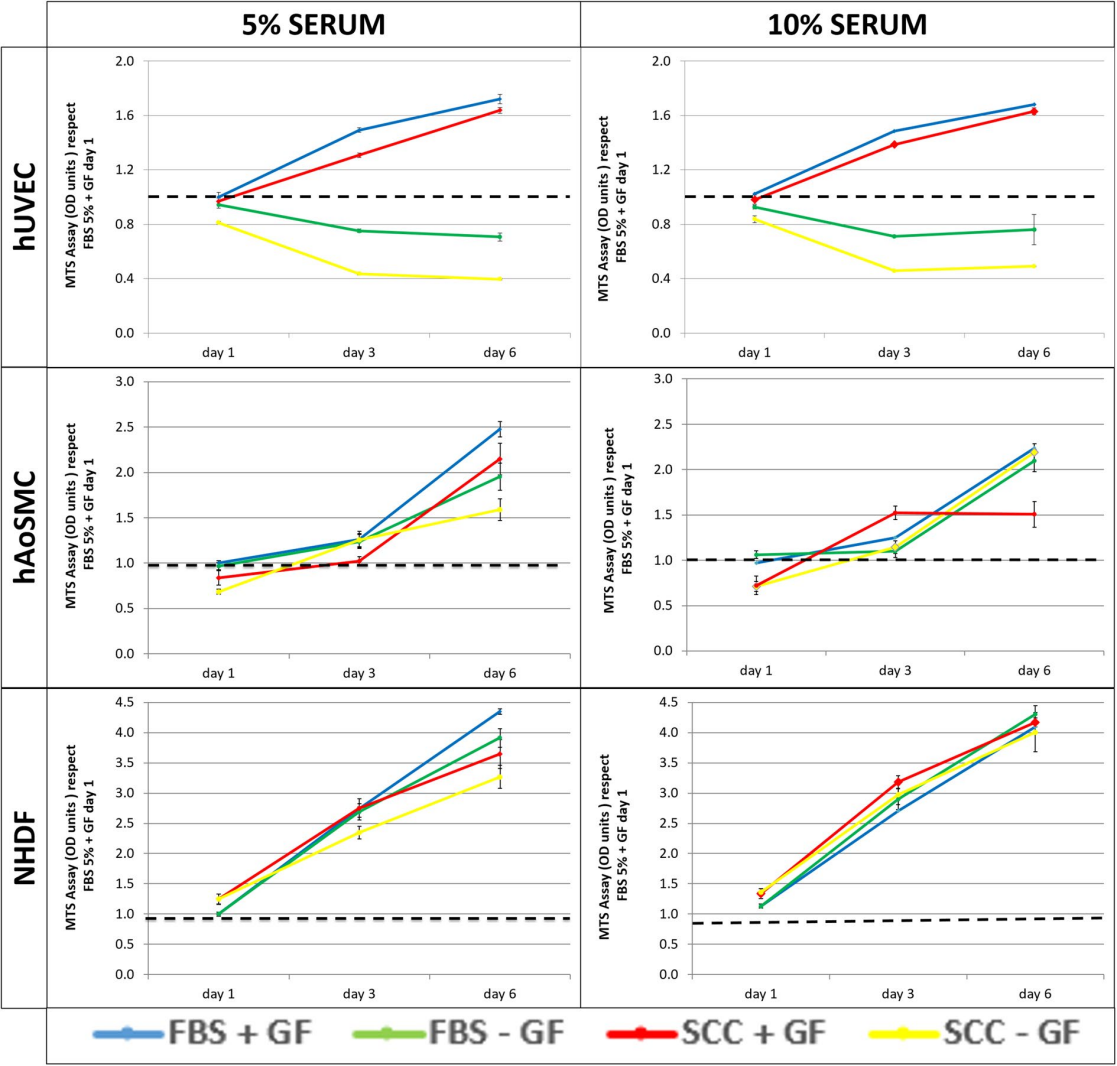
Viability assay of different cell types growing in media supplemented with different concentrations of FBS and SCC in presence or absence of GF after 6 days of culture. The first column corresponds to 5% of FBS or SCC and the second to 10% of FBS or SCC. Results are normalized to the MTS value at day 1 for cells supplemented with growth factors and FBS at the concentration recommended by the provider. Results are presented as mean ± standard deviation obtained from three independent values per group. One-way analysis of variance was performed comparing means with Tukey’s post-test. All assays comparisons were made by correlation and linear regression analysis. Differences were considered significant if p < 0.05

Viability assay was conducted using a tetrazolium salt assay, specifically MTS ((3-(4,5-dimethylthiazol-2-yl)-5-(3-carboxymethoxyphenyl)-2-(4-sulfophenyl)-2H-tetrazolium)) during the first 6 days of culture. Results summarized in Fig. 2 show the daily MTS values normalized to the MTS value at day 1 for cells cultured as recommended by the provider (5% FBS + GF). Growth factors (GF) deprivation caused a significant decrease in MTS signal in hUVEC. Only hAoSMC culture with 5% SCC without GF showed lower MTS signal than the other conditions. In contrast, the absence of GF in NHDF cultures did not cause any significant difference in cell proliferation. Interestingly, in the presence of growth factors, no significant differences were observed between cells cultured with SCC supplemented media and those cultured with FBS supplemented media in any cell type. All cells studied proliferated at similar rates regardless of the supplement applied. NHDF growth kinetics were almost independent of sera type and concentration.

### Cellular structure

**Fig. 3:**
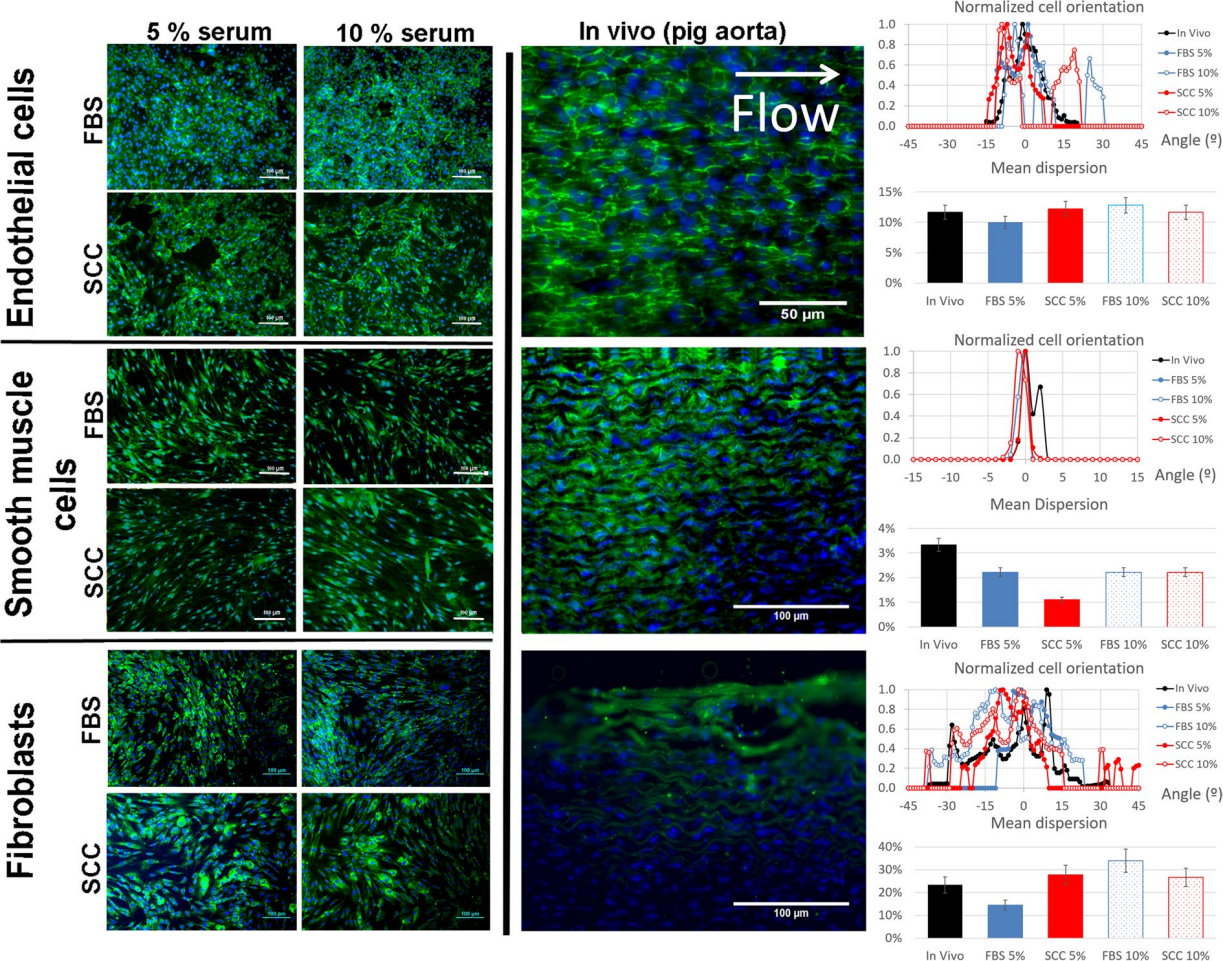
Analysis of cell structure by immunofluorescence. In terms of cell structure, CD31 was stained for endothelial cells, actin α smooth muscle for smooth muscle cells and collagen I for fibroblasts. Specific proteins are stained in *green* and nuclei in *blue*. Cellular orientation distribution was measured using Fiji’s plugin “Orientation J Distribution”. The Gaussian distribution of cellular orientations was centered on 0 and all values were divided by each case’s maximum value in order to get a normalized orientation value between 0 and 1. The mean dispersion percentage was calculated as the angle range that contained 80% of the orientations, divided by 180°

Cellular structure is a determinant of the cellular phenotype. We tested whether SCC modified cellular morphology, comparing it to that of cells cultured in FBS-supplemented medium. We also compared our cultures with in vivo samples from the aorta of a Yorkshire pig. Figure 3 shows the morphological structure of cells supplemented with different levels of FBS or SCC and the comparison with the phenotype observed in a porcine aorta. The adjacent graphics show the cellular orientation dispersion in the in vivo example compared with the dispersion of in vitro cell cultures.

hUVEC cultured in all conditions showed homogeneous expression of platelet/endothelial cell adhesion molecule 1 (PECAM-1 or CD31) in their membranes and especially at the intercellular junctions. Endothelial cells cultured in FBS and SCC were disposed in a classic cobblestone display. Intercellular connectivity through CD31 bridges was maintained in all cases. In the porcine model, endothelial cells presented a paved structure aligned to flow [9]. The in vivo orientation analysis shows two peaks, coherent with a rectangular shape. Similarly, endothelial cells cultured in vitro with FBS or SCC present a similar distribution. Indeed, the mean dispersion of orientations was around 10% in all cases. hAoSMC cultured in all conditions displayed uniform expression of α-smooth muscle actin. Actin filaments were aligned even when multilayers were formed. In the porcine aorta, an extensive multilayer of smooth muscle cells can be observed, with cells clearly aligned and elongated perpendicular to flow. The in vivo orientation analysis shows a narrow peak, coherent with cells with an elongated shape and clear alignment. Smooth muscle cells cultured in vitro with FBS or SCC present an analogous morphology, and the mean dispersion of orientations was below 4% in all cases. No differences in collagen I expression were observed between NHDF cultured in FBS- or SCC-supplemented medium. Collagen I fibers bundled to ensure cohesion and continuity of the fibroblast multilayer, both in the cultures and the naïve aorta. Of note, dermal and adventitial have many phenotypical differences. However, they both create supportive tissue by building a protein network, using collagen fibers and fibronectin amongst other molecules. As fibroblasts do not align but create a support tridimensional matrix, their spatial distribution is much sparser. Our orientation analysis revealed a broad plateau, both in vitro and in vivo, with mean dispersion ranging between 15 and 35%.

In conclusion, cells incubated with FBS- and SCC-supplemented media exhibited the same morphological endogenous markers than in vivo and a similar spatial distribution.

## Cellular activity

**Fig. 4:**
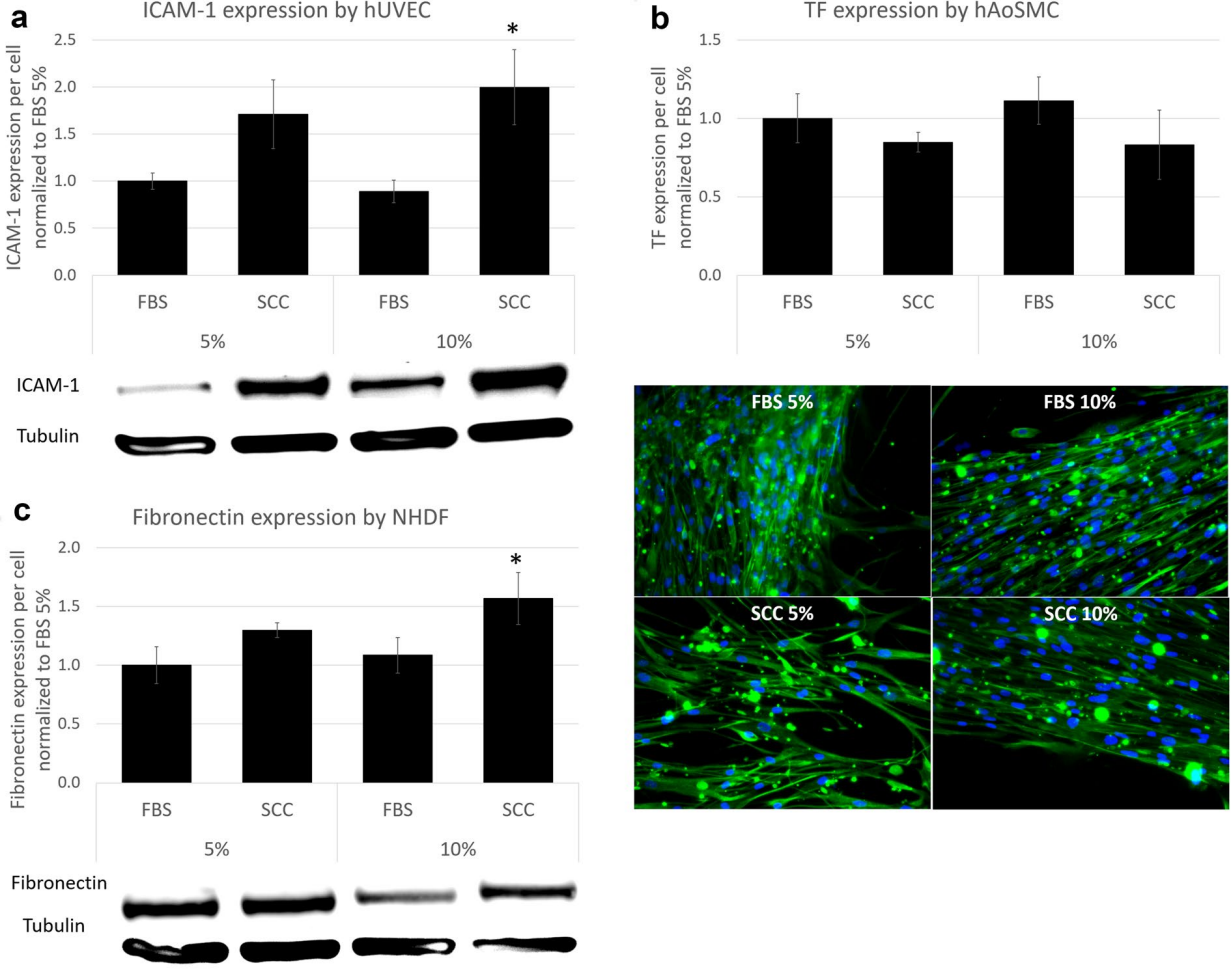
Cell activity analysis. Comparison of protein expression between cells supplemented with FBS and SCC at different levels. **a** ICAM-1 expression was analyzed by Western Blotting after the addition of TNF-α. **b** Tissue factor expression in injured human aortic smooth muscle cells was evaluated by immunofluorescence. The fluorescence intensity (*green*) was measured at the edge of the injury and divided by the number of cells. **c** Fibronectin expression in human normal dermal fibroblasts was analyzed by Western Blotting. All results are normalized to the value of FBS 5% and presented as mean ± standard error of mean as obtained of three independent values per group. Nonparametric Kruskal–Wallis test, followed by a Scheffé posthoc analysis was performed, and differences were considered significant if p < 0.05

A marker of cellular activity relevant to each studied cell type was chosen and examined at different FBS and SCC concentrations. Interestingly, the cellular reactivity of studied cells was significantly different between the proposed serum and FBS, the standard used in research. Inflammatory response of hUVEC was measured by western blot after addition of tumor necrosis factor alpha (TNF-α) to stimulate intercellular adhesion molecule 1 (ICAM–1) expression (see Fig. 4a). Pro-coagulant response to injury of smooth muscle cells was evaluated by quantitative immunofluorescence of tissue factor (TF) expression (see Fig. 4b). NHDF extracellular matrix synthesis was detected measuring fibronectin expression using Western Blot (see Fig. 4c). In vivo, endothelial cells are responsible to form the vascular endothelium, a semipermeable barrier responsible for regulating the exchange between blood and peripheral tissues. Addition of the inflammatory cytokine TNF-α to the culture medium provoked a significant increase of ICAM-1 expression in FBS- and SCC-supplemented media in all cases (data obtained without TNF-α is not shown). Results of samples treated with TNF-α are expressed as ICAM-1 expression divided by tubulin expression and normalized to the results obtained in cells cultured in medium supplemented with 5% FBS. ICAM-1 expression after TNF–α stimulation was up to two-fold higher in cells supplemented with SCC compared to cells supplemented with FBS (p < 0.05) (Fig. 4a). One may infer that SCC enhances ICAM-1 expression in hUVEC. This result is very relevant in case of transplantation of endothelial cells in novel arterial replacements [10]. The expression of ICAM-1 could enhance endothelial progenitor cells recruitment and angiogenesis, favoring revascularization of the implant and hence increasing the chance of success.

Exposure of tissue factor (TF) present in smooth muscle cells to the blood torrent triggers the coagulation cascade. Overexposure of TF serves as a key precipitant of injury-related local clot formation and can lead to thrombosis, a potentially lethal event. In animal models of balloon arterial injury, TF is rapidly induced in smooth muscle cells. In our experiments, we injured hAoSMC in vitro and analyzed TF after injury. TF was overexpressed in cells surrounding the injury area, and such cells migrated towards the denuded, cell-free regions. Results are expressed as TF expression in injured cells relative to TF expression in non-injured cells. The results presented in Fig. 4b show that TF expression was not different between sera concentrations nor origin. In all cases, injured hAoSMC increased their TF expression between 30 and 50% when compared to their intact neighbors. We observed that TF expression bump after injury was marginally higher in hAoSMC cultured with FBS than in SCC, but not significantly. SCC did not affect this two-edged sword defense mechanism in hAoSMC.

In fibroblasts, activity is given by the ability to build extracellular matrix (ECM). Fibronectin, which binds to integrins, collagen, fibrin and heparin sulfate proteoglycans, plays a major role in cell adhesion, growth, migration and differentiation. Figure 4c shows that fibronectin was slightly overexpressed in fibroblasts cultured with SCC medium, but only significantly (p < 0.05) when comparing 10% SCC to 10% FBS. These results indicate that SCC promotes extracellular matrix formation by fibroblasts as powerfully as FBS at 5% serum concentration and better at 10%. These differences may be due to different concentration of low molecular weight molecules. These molecules could alter ECM protein deposition in fibroblasts and depleting SCC from them could be an inherent advantage to the product.

## Conclusions

We have shown how SCC, a human plasma derived supplement SCC is able to sustain growth, morphology and function of human endothelial cells, smooth muscle cells and fibroblasts, equally or even better than fetal bovine serum. As a xeno-free product, GMP-compliant and ensuring lot to lot consistency, the introduction of SCC at early stages of development of advanced cell therapies may ease fulfillment of safety and efficacy requirements prior to clinical studies.
